# Food provisioning increases the risk of injury in a long-lived marine top predator

**DOI:** 10.1098/rsos.160560

**Published:** 2016-12-21

**Authors:** Fredrik Christiansen, Katherine A. McHugh, Lars Bejder, Eilidh M. Siegal, David Lusseau, Elizabeth Berens McCabe, Gretchen Lovewell, Randall S. Wells

**Affiliations:** 1 Cetacean Research Unit, School of Veterinary and Life Sciences, Murdoch University, Murdoch, Western Australia 6150, Australia; 2 Sarasota Dolphin Research Program, Chicago Zoological Society, c/o Mote Marine Laboratory, Sarasota, FL, USA; 3 Institute of Biological and Environmental Sciences, University of Aberdeen, Aberdeen AB24 2TZ, UK; 4 Mote Marine Laboratory, Sarasota, FL, USA

**Keywords:** anthropogenic disturbance, behaviour, dolphin, human exposure, Sarasota, wildlife management

## Abstract

Food provisioning of wildlife is a major concern for management and conservation agencies worldwide because it encourages unnatural behaviours in wild animals and increases each individual's risk for injury and death. Here we investigate the contributing factors and potential fitness consequences of a recent increase in the frequency of human interactions with common bottlenose dolphins (*Tursiops truncatus*) in Sarasota Bay, Florida. A rising proportion of the local long-term resident dolphin community is becoming conditioned to human interactions through direct and indirect food provisioning. We investigate variables that are affecting conditioning and if the presence of human-induced injuries is higher for conditioned versus unconditioned dolphins. Using the most comprehensive long-term dataset available for a free-ranging bottlenose dolphin population (more than 45 years; more than 32 000 dolphin group sightings; more than 1100 individuals), we found that the association with already conditioned animals strongly affected the probability of dolphins becoming conditioned to human interactions, confirming earlier findings that conditioning is partly a learned behaviour. More importantly, we found that conditioned dolphins were more likely to be injured by human interactions when compared with unconditioned animals. This is alarming, as conditioning could lead to a decrease in survival, which could have population-level consequences. We did not find a significant relationship between human exposure or natural prey availability and the probability of dolphins becoming conditioned. This could be due to low sample size or insufficient spatio-temporal resolution in the available data. Our findings show that wildlife provisioning may lead to a decrease in survival, which could ultimately affect population dynamics.

## 1. Introduction

Wildlife provisioning, intentional or inadvertent, plays an important role in shaping animal communities, food webs and ecosystems [1]. The occurrence of wildlife provisioning is increasing globally as a consequence of an increase in human food waste production and the frequency of human–wildlife interactions [1–3], both the result of human population growth. In some contexts, provisioning wildlife with additional food resources can have positive effects on individual survival and reproductive success [4,5] leading to an increase in population density [6–8], and even helping the recovery of threatened species [9]. However, provisioning often has serious short-term and long-term negative effects on both animals and humans (see [10,11] for review).

The short-term negative effects of wildlife provisioning include changes in activity budgets [12], an increase in field metabolic rates [13], a reduction in home range sizes [5,6] and an increase in both intra- and interspecies aggression [14,15]. In the long-term, animals can become conditioned to human interaction through food provisioning (hereinafter referred to as ‘conditioned’) [16,17], with animals associating humans with food, and therefore seeking close-up interactions with humans [14,18,19]. Such close-up interactions can have harmful effects on the conditioned animals, by increasing the risk of injury [19,20], disease [21] and even death [22]. Further, food provisioning can lead to wildlife becoming aggressive towards humans [19,23], sometimes leading to injuries [23] and the death of humans [24]. Such tragic interactions can, in turn, lead to the provisioned animal being destroyed by authorities [24]. In the light of the risks of injury and death to both animals and humans from food provisioning, understanding the factors that lead to animals becoming conditioned, how conditioning spreads through a population and what the fitness consequences are for conditioned wildlife, is crucial for wildlife managers to regulate such interactions.

Bottlenose dolphins (*Tursiops* spp.) around the world are subject to food provisioning from humans, including incidental (food discard and depredation), illegal (unregulated food handouts) and regulated provisioning (feeding programmes) [16,17,25,26]. Bottlenose dolphins are commonly found in coastal areas where humans participate in water-related commercial, recreational and tourism activities, which increase the frequency of human–dolphin interactions. Food provisioning of wild bottlenose dolphins can lead to changes in behaviour [16,27] and increasing intraspecific aggression [28]. Regular close-up interactions with boats and fishing gear also put cetaceans at risk of injuries from boat strikes and entanglement in fishing gear [20], as well as ingestion of inappropriate food items and fishing gear [26,27]. Close-up interactions also facilitate the transmission of disease between humans and dolphins [29]. In a number of instances, conditioned dolphins have died as a consequence of injuries caused either directly or indirectly by humans or from ingesting fishing gear [28,30,31]. Dependency on food provisioning has also been reported to reduce reproductive success in dolphins, by increasing first-year mortality in calves [32,33]. Further, there are several documented cases where conditioned dolphins have started to show aggression towards humans [25], sometimes leading to attacks and injuries on humans [26,28,34]. In one case, a swimmer was killed by a conditioned dolphin after having provoked it [34].

With the frequency of human–dolphin interactions increasing globally as a consequence of increasing coastal development [35,36], a better understanding of the causes and consequences of provisioning is necessary to inform management decisions about how to regulate such interactions. In order for conditioning to occur, some level of exposure to human activities is necessary. Donaldson *et al.* [16] found that conditioning in Indo-Pacific bottlenose dolphins (*Tursiops aduncus*) in Cockburn Sound, southwest Australia, increased as a function of exposure to human activities and also by dolphins associating more with already conditioned animals. The latter suggests that conditioning is at least partly a socially learned behaviour in bottlenose dolphins which is likely to speed up the rate at which animals in a population become conditioned. Prey availability is another variable that may influence the probability of dolphins seeking food from anthropogenic sources; however, no study to date has investigated this.

This study investigates conditioning in common bottlenose dolphins (*Tursiops truncatus*) subject to illegal and incidental food provisioning by humans in Sarasota Bay, Florida. Over the last several decades, there has been an increase in the frequency of adverse human–dolphin interactions throughout the coastal waters of this region [26]. This has become a major management and conservation concern because of the potential for human–dolphin interactions to lead to unnatural foraging behaviours such as begging, depredation and scavenging, which in turn might lead to human-induced injuries or mortalities for conditioned animals [30,37,38]. In the USA, food provisioning and the harassment of dolphins by humans are prohibited as ‘takes’ under the federal Marine Mammal Protection Act. Although previous studies in Sarasota Bay have investigated the effects of food provisioning on dolphins [26,27,31], the complexity of the problem and the suite of variables potentially contributing to human–dolphin interactions (and their spatial and temporal components) have made this very challenging. Here, we capitalize on the largest long-term dataset (more than 45 years) on free-ranging bottlenose dolphins to investigate the rate of increase in conditioning in this population, and the variables that are driving this increase. We further examine the potential fitness consequences of conditioning on dolphins, by comparing the relative proportion of human-induced injuries between conditioned and unconditioned animals.

## 2. Material and methods

### 2.1. Study site

**Figure 1.**
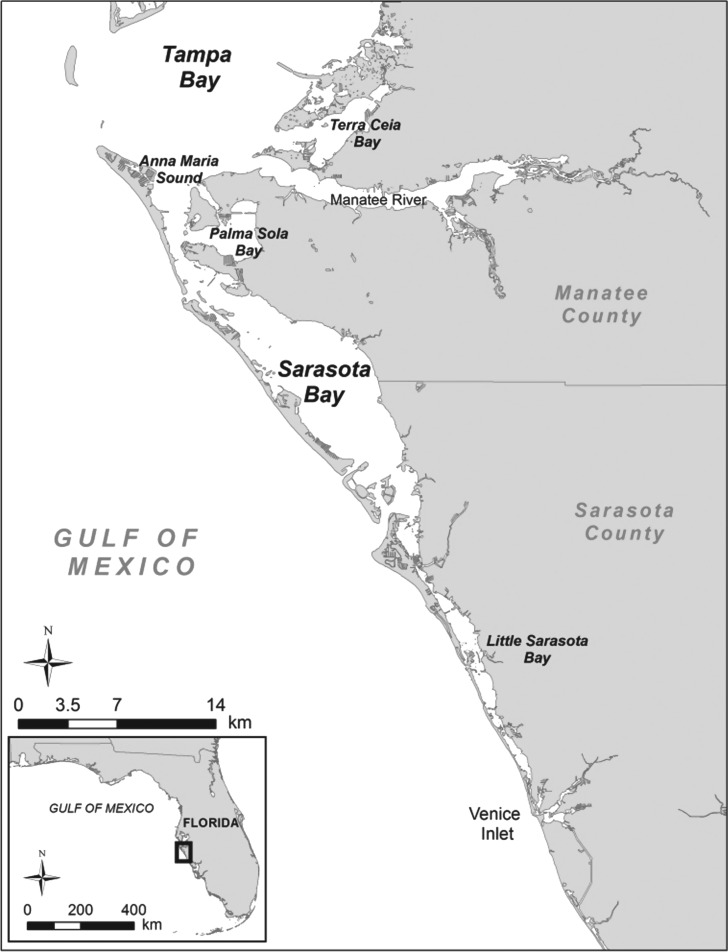
Map of the Sarasota Bay study area, which runs from the southern edge of Tampa Bay to Venice Inlet on the central west coast of Florida.

Sarasota Bay, Florida is home to the long-term Sarasota Dolphin Research Program (SDRP), whose investigators have studied a multi-generational resident bottlenose dolphin community since 1970 and provided background knowledge on the population's social structure, life history, behaviour, health and physiology, and ecology [39,40]. As of 2014, the resident dolphin community of approximately 160 dolphins spanned at least four concurrent generations from newborns up to 64 years in age, residing in inshore waters on the central west coast of Florida from southern Tampa Bay to Venice Inlet and up to several km into the Gulf of Mexico (figure 1) [40]. This range encompasses a variety of shallow-water habitats along approximately 40 km of coastline. Based on long-term observations and periodic health assessments using temporary capture–release efforts, 96% of the dolphins using Sarasota Bay and associated waters on a regular basis are individually identifiable, and more than 90% of resident animals are of known age, sex, maternal lineage, paternity or a combination of these. On average, one-third of all resident dolphins that have died or permanently disappeared each year are recovered as carcasses [41], providing supplementary information to ongoing field efforts.

The Sarasota Bay region is also home to a growing human population, where dolphins are increasingly exposed to interactions with recreational fishing, boating and coastal tourism operations. Within the home range of the Sarasota resident dolphin community, which includes both Sarasota and Manatee counties (figure 1), the human population has more than tripled and the number of registered boats has quadrupled since 1970 [27], with approximately 40 000 registered boats and over 50 000 recreational saltwater fishing licences active in 2014. There also continues to be limited inshore commercial fishing activity following a state-wide commercial net fishing ban implemented in 1995, with primarily crab fisheries using trap/pot gear actively within the study region since that time.

Adverse human–dolphin interactions in this region take several different forms, but primarily involve interactions between dolphins and recreational anglers or boaters. Illegal direct provisioning of animals has occurred throughout the region, with a concentrated hot spot of begging and provisioning observations in the southern portion of the range, focused around a small number of individuals habituated to seeking food from humans [26,27]. Incidental provisioning in connection with recreational fishing activity is more frequent, with a growing number of animals observed patrolling, scavenging and depredating bait and/or catch from hook and line anglers at a rate of up to approximately 20% of the resident community in any given year [27]. Some incidental provisioning has also come in the form of dolphins interacting with fixed fishing gear (i.e. crab traps and pots) which can concentrate prey. Injuries to Sarasota Bay dolphins (and in some cases humans) have resulted from these activities, with approximately 35% of those observed with human-related injuries serious enough to have likely contributed to death or requiring rescue interventions to ensure survival (K.A.M. 2014, personal observation).

### 2.2. Data collection

**Figure 2.**
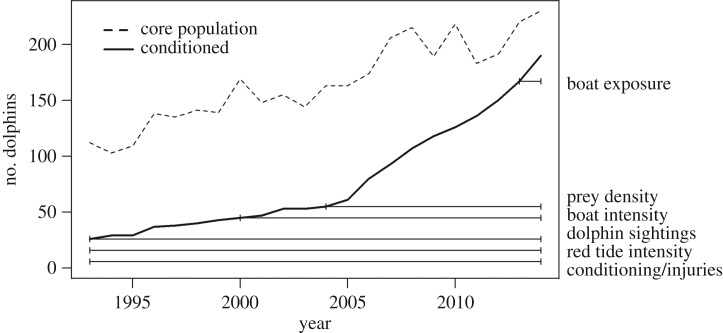
The cumulative number of conditioned dolphins (solid line) observed over the study period (1993–2014). The core dolphin population size (dashed line), representing animals seen during at least four months or two seasons of the year within the core study area based on all field effort is shown for comparison. The horizontal bars indicate the time periods covered by the different datasets used in this study. Observe that the cumulative number of conditioned dolphins does not account for conditioned animals that died during the study.

To investigate the effects of food provisioning on dolphins in Sarasota Bay, this study used a number of datasets that varied in length of time period covered (figure 2 and also see the electronic supplementary material).

Dolphin identity (ID) and behavioural data were collected year-round on a monthly basis in Sarasota Bay between 1993 and 2014 (earlier data were collected on different schedules and are not included in analyses here) [40]. Standard photographic identification surveys and mark–recapture techniques [42] were used to identify individual dolphins and determine the proportion of individuals interacting with humans. The age and sex of dolphins were also recorded as part of the long-term research programme (initiated in 1970). Ages of free-ranging dolphins were either determined from longitudinal sighting histories of individuals known since birth [39] or estimated from examination of growth layer groups in teeth extracted during necropsy or under local anaesthesia during health assessment capture–release efforts [43]. Sexes were determined either by direct observation or examination of the genital region, genetics or for some females, repeated observation with a dependent calf [39]. The presence of human-related body injuries, including fishing gear entanglement, external hooking or ingestion and boat strike injuries, were noted when observed either directly in the field, during capture–release efforts for life-history, rescue operations or health assessments, or upon necropsy evaluation (see the electronic supplementary material).

Temporal and spatio-temporal data on human activities (fishing, recreational boaters, and tourism businesses) were recorded during 2000–2014 and 2013–2014, respectively (see the electronic supplementary material). Data on dolphin prey abundance were collected during an ongoing multi-species fish survey in Sarasota Bay [44] from 2004 to 2014 (see the electronic supplementary material). *Karenia brevis* cell abundances were used as a proxy for measuring red tide bloom intensities. *Karenia brevis* samples were collected, and the data recorded during 1987–2014 (see the electronic supplementary material).

Research on free-ranging dolphins was conducted under a series of US National Marine Fisheries Service Scientific Research Permits (most recently no. 15543) issued to R.S.W., and under annual IACUC approvals through Mote Marine Laboratory (most recently 15-11-RW1). Fish sampling was performed under a series of Florida Fish and Wildlife Conservation Commission Special Activity Licenses (most recently SAL-13-0809-SR) issued to E.B.M. and under annual IACUC approvals through Mote Marine Laboratory (most recently 15-11-RW2).

### 2.3. Conditioning

A dolphin was classified as conditioned from the first time it was observed interacting with humans and performing one of the following behaviours indicative of conditioning [17,16]: patrolling, scavenging, depredation, begging, provisioning and fixed gear interactions (see the electronic supplementary material, table S1 for definitions of behaviours). Dolphins that did not display any of these behaviours during the study period were termed ‘unconditioned’.

For conditioned dolphins, the proportion of time engaged in human–dolphin interactions (no. human–dolphin interactions/no. sightings) per year since becoming conditioned was investigated. To avoid bias from small sample size (i.e. few sightings), only conditioned animals that had been sighted on at least 10 separate days per year for at least 2 years since becoming conditioned were included in the analysis. To investigate the amount of individual variation in the rate of human–dolphin interactions as a function of time since conditioning, a linear model was fitted for each individual and the slope parameter from each model was plotted in a frequency histogram.

### 2.4. Variables affecting the probability of conditioning

We developed generalized linear models (GLMs) in R to determine which variables best explained conditioning in bottlenose dolphins in Sarasota Bay. The covariates used in the model were exposure to human activities (EXP), the coefficient of association (COA) with already conditioned animals, age and sex.

EXP was estimated for each individual dolphin for 2013 and 2014 by overlapping individual dolphin encounter probabilities with human intensity data in the study area (see the electronic supplementary material) [45,46]. Dolphin encounter probabilities were estimated using spatially explicit capture–recapture (SECR) models, whereas human intensity data (density of boats, crab pots and fishing line) were recorded during dolphin surveys (see the electronic supplementary material). EXP represents the average per minute probability of an animal being exposed to any type of human activity (boats, crab pots and fishing lines) in a given year. COA with conditioned dolphins was estimated using the method developed by Donaldson *et al.* [16], who used the half-weight association index [47] to quantify the number of times two individual dolphins were sighted together in a year relative to how often they were sighted separately (see the electronic supplementary material). COA provides a relative measure of association with conditioned animals, ranging from 0 (no association with conditioned animals) to 1 (association only with conditioned animals). Only dolphins that had been sighted on at least 10 days during the study period (*n* = 604 individuals) were included in the analysis. To investigate the time period over which conditioning is transmitted between conspecifics, COA was estimated over different time periods, ranging from 1 to 5 years.

Similar to Donaldson *et al.* [16], we assumed that EXP and COA in a given year would affect the probability of conditioning in dolphins in the following year (e.g. conditioning in 2014 being affected by EXP and COA in 2013). This, however, restricted our analysis using all four explanatory variables to investigate conditioning in 2014 only (EXP data only existed for 2013 and 2014). To increase statistical power, we also ran a separate analysis on a subset of the data where EXP was excluded from the analyses.

A GLM with a binomial distribution (conditioning as a binary variable) and logit link function was fitted to the data. In the model selection process, covariates and interactions between covariates were added sequentially to the null model based on biological explanation. Collinearity (high correlation) between the explanatory variables in the model was investigated by estimating the variance inflation factor (VIF), with an upper threshold value of three indicating collinearity. Overdispersion was tested for each model by dividing the residual deviance with the residual degrees of freedom, with a ratio value (dispersion parameter, *φ*) above one indicating overdispersion (the mean of the variance is larger than the mean). The best-fitting model was selected using Akaike's information criterion (AIC).

### 2.5. Variables affecting the number of conditioned dolphins

To investigate which variables best explained the observed number of conditioned dolphins between years, we used a GLM with a Poisson distribution (number of conditioned dolphins as a count variable) and a log link function. The explanatory variables examined were boat intensity (the annual number of registered boats in Manatee County and Sarasota County), COA with conditioned dolphins (the average COA with conditioned animals for all unconditioned animals for a given year), prey abundance (catch-per-unit-effort of selected dolphin prey species) and red tide intensity (number of weeks per year with *K. brevis* concentrations above fish kill levels, more than 100 000 cells per litre; see the electronic supplementary material). For the COA estimates, only dolphins that had been sighted at least 10 times in a given year were included. To investigate potential lags in the relationship between prey density and conditioning, we modelled the relationship between the two variables based on values of prey density both in the current and the previous year. The same was done for red tide intensity and COA.

Model selection was based on AIC, with covariates and interactions between covariates being added sequentially to the null model based on biological explanation. Collinearity was investigated using VIF, and overdispersion was tested for each model by dividing the residual deviance with the residual degrees of freedom. To account for overdispersion in the models, the standard errors were corrected using a quasi-GLM model where the variance is given by *ϕ* × *μ*, where *μ* is the mean and *ϕ* the dispersion parameter.

### 2.6. Fitness consequences of conditioning

To examine the potential fitness consequences of conditioning in dolphins, we examined the relationship between the occurrence of human-induced injuries and conditioning. A GLM with a binomial distribution (injury as a binary variable) with a logit link function was fitted to the data. Because the probability of an animal acquiring an injury is likely to increase over time as the animal gets older, we included age as a covariate in the model. Sex was also included as a covariate. Collinearity between the explanatory variables in the model was again investigated using VIF and overdispersion was tested by dividing the residual deviance with the residual degrees of freedom. The best-fitting model was selected using AIC.

## 3. Results

### 3.1. Sampling effort

In total, 32 521 dolphin groups were sighted during 1993–2014 in the study area. Although the number of sightings was high throughout the study period, the number of sightings was generally higher in the summer, particularly in 2000, 2001, 2007 and 2008 (see the electronic supplementary material, figure S1).

### 3.2. Conditioning

During 1993–2014, a total of 1142 individual dolphins were identified. Of these, 110 (9.6%) were confirmed dead before the end of the study period. The number of conditioned dolphins increased over the study period (figure 2). In total, 25 dolphins were conditioned prior to the start of this study in 1993. Before 2000, the number of conditioned dolphins was fewer than 50; however, in the following years, this number increased rapidly, particularly after 2005. In 2008, more than 100 dolphins were conditioned, and at the end of the study period (i.e. 2014), 190 animals (16.6% of all identified individuals) had become conditioned (figure 2). Of the conditioned animals of known sex (78.9%), the ratio of males to females was 1 : 1.

**Figure 3.**
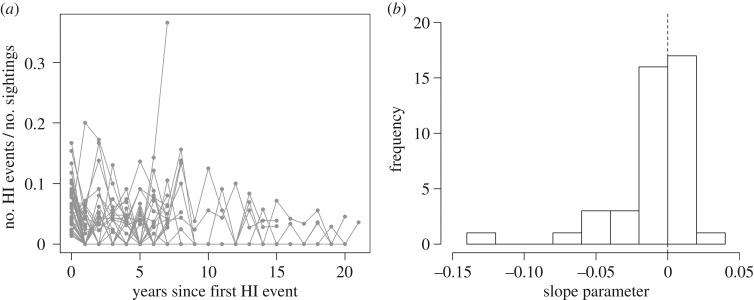
(*a*) Proportion of time conditioned dolphins engaged in human–dolphin interactions (HI events) as a function of years since first HI event. (*b*) Frequency distribution of slope parameters from the linear models investigating the relationship between proportion of HI events as a function of years since first HI event. The dashed vertical line indicates the cut-off point between negative and positive rates of change in proportion of HI events over time. *n* = 42 conditioned dolphins.

After becoming conditioned, the proportion of time that dolphins engaged in human–dolphin interactions varied between individuals and also over time (figure 3). On average, dolphins were observed to engage in human–dolphin interactions in 3.5% of the sightings (s.d. = 2.6, median = 2.8, min = 0.3, max = 10.7). Of the 42 conditioned dolphins investigated (individuals with more than 10 sighting per year since becoming conditioned), 42.9% (*n* = 18) showed an increase in the proportion of time engaged in human–dolphin interactions over time, whereas the remaining 57.1% (*n* = 24) showed a decrease (figure 3*b*).

### 3.3. Variables affecting the probability of conditioning

**Figure 4.**
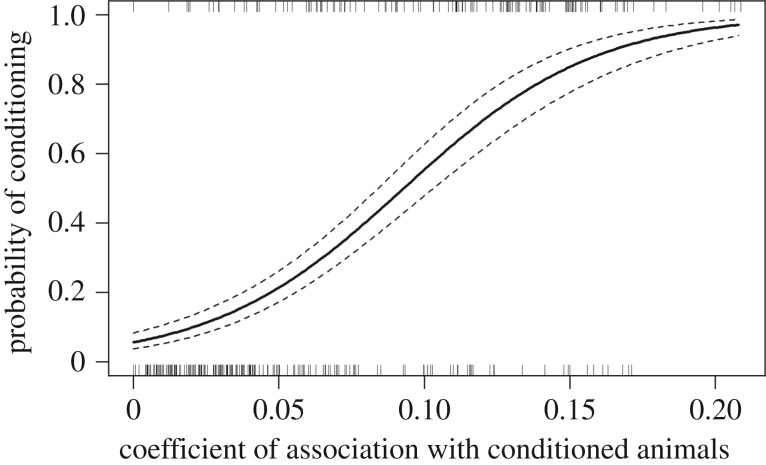
Probability of conditioning in bottlenose dolphins as a function of the coefficient of association (COA) with already conditioned animals during the preceding 2 years. The solid line represents the fitted values of the generalized linear model. The dashed lines represent 95% CIs. The distribution of COA values for conditioned and unconditioned dolphins are shown by the top and bottom rug plots, respectively. *n* = 524.

When including all four explanatory variables (EXP, COA, age and sex) in the GLM, none had a significant effect on the probability of dolphins becoming conditioned. The boat exposure data, however, limited the analyses to a relatively small dataset of conditioned animals (*n* = 42) in 2014. When excluding EXP from the model, the dataset could be expanded (*n* = 187 dolphins for which COA, age and sex was known). When analysing this subset of the data, we found a significant effect of COA on the probability of dolphins being conditioned (*z* = 7.19, *p* < 0.001, *n* = 187), with COA explaining 29.9% of the deviance (pseudo-*R*^2^) in the data. Sex and age did not have a significant effect on conditioning. There was no collinearity between the explanatory variables used in the GLM and no sign of overdispersion (*φ* = 1.08). The time period over which COA was estimated did not change the relationship between COA and conditioning substantially (see the electronic supplementary material, figure S2), although the best model fit was achieved when COA was estimated over 2 years prior to conditioning (*z* = 11.86, *p* < 0.001, *n* = 524). The best-fitting model explained 35.9% of the deviance (pseudo-*R*^2^) in the data (figure 4). The estimated dispersion parameter showed no sign of overdispersion (*φ* = 0.94).

### 3.4. Variables affecting the number of conditioned dolphins

**Figure 5.**
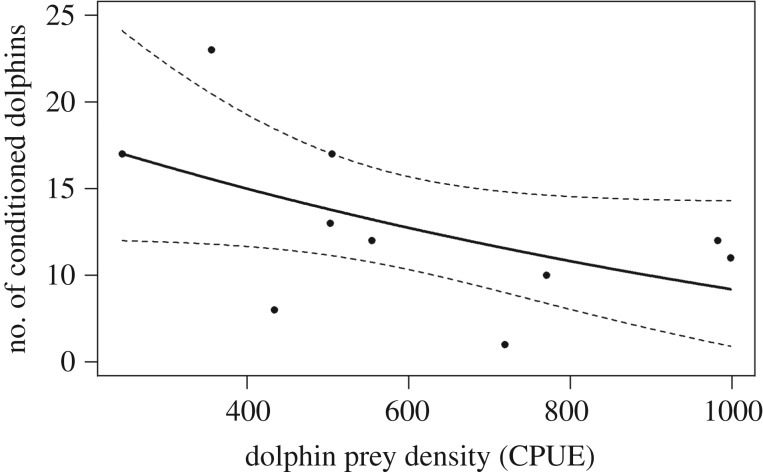
Number of conditioned dolphins per year as a function of dolphin prey density (catch-per-unit-effort (CPUE)) in the area in the previous year. The solid line represents the fitted values of the quasi-generalized linear model. The dashed lines represent 95% CIs. *n* = 10 years.

All four explanatory variables (boat intensity, mean COA with conditioned animals, dolphin prey density (catch-per-unit-effort; CPUE) and red tide intensity) fluctuated over the study period (see the electronic supplementary material, figure S3). None of the four explanatory variables, with or without lags, had a significant effect on the number of conditioned dolphins observed per year (*n* = 10 years including all four variables). However, when modelling the number of conditioned dolphins as a function of dolphin prey density (CPUE) in the preceding year, a weak negative relationship was found (*χ*^2^ = 4.73, *p* = 0.068, *n* = 10, figure 5). The quasi-GLM model explained 28.4% of the deviance (pseudo-*R*^2^) in the data and the dispersion parameter (*ϕ*) was taken to be 1.42.

### 3.5. Fitness consequences of conditioning

**Figure 6.**
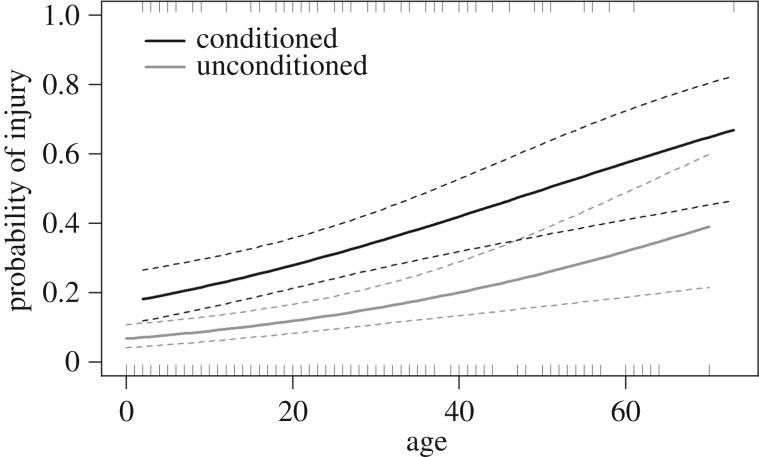
Probability of injury as a function of age for conditioned (black solid line) and unconditioned (grey solid line) bottlenose dolphins. The solid lines represent the fitted values of the generalized linear model. The dashed lines represent 95% CIs. The distribution of age values for conditioned and unconditioned dolphins are shown by the top and bottom rug plots, respectively. *n* = 404.

Of the 404 dolphins included in the analysis, 75 (18.6%) were injured from human–dolphin interactions. Conditioning and age both had a significant effect on the probability of dolphins being injured through human–dolphin interactions (figure 6). A higher proportion of conditioned animals were injured compared with unconditioned animals (*z* = 3.90, *p* < 0.001, *n* = 404; figure 6). As expected, the probability of animals being injured also increased with the age of the animals (*z* = 3.93, *p* < 0.001, *n* = 404; figure 6). The full model explained 9.1% of the deviance (pseudo-*R*^2^) in the data. The estimated dispersion parameter showed no signs of overdispersion (*φ* = 0.99). There was no significant interaction between age and conditioning (*z* = −0.04, *p* = 0.965), and there was no difference between sexes in the probability of being injured (*z* = 1.01, *p* = 0.312, *n* = 241).

## 4. Discussion

The number of bottlenose dolphins conditioned to human interactions through food provisioning is increasing rapidly in Sarasota Bay. This trend is concurrent with earlier findings by Powell & Wells [27], who found that the rate of human–dolphin interactions is increasing in the area. However, while the proportion of conditioned animals has nearly tripled in the last 10 years, our findings show that only a small proportion of conditioned dolphins interacted frequently with humans. We found no bias in the sex of conditioned dolphins, in difference to Finn *et al.* [17], who found that conditioned dolphins in Cockburn Sound, southwest Australia, were predominantly males. Conversely, in Monkey Mia, Western Australia, management decisions have restricted provisioning to females only [32]. The increase in number of conditioned dolphins in Sarasota Bay could not be attributed to an increase in boat intensity in the area. Similarly, although association with conditioned animals was found to influence the probability of a dolphin becoming conditioned, when looking at the total number of conditioned dolphins in a given year, the average COA with conditioned animals had no effect (see §3.4). A possible explanation for this discrepancy could be that conditioning in dolphins is driven by very strong associations with a small number of conditioned dolphins, rather than the overall likelihood of an animal encountering or associating with conditioned dolphins. Social network analysis could be used to investigate this further. Although not statistically significant, we did find a weak negative relationship between the number of conditioned dolphins in a given year and the density (CPUE) of dolphin prey species in the previous year. A reduction in natural prey could force dolphins to interact more with humans in order to obtain supplemental feeding from such interactions. The rapid increase in conditioning beginning in 2005–2006 may have resulted at least in part from an especially severe red tide event in 2005 which decimated overall fish abundance, including many key dolphin prey species, made significant changes to estuarine fish community structure, and shifted resident dolphin behaviour and association patterns temporarily [44,48]. We were not able to find a statistically significant relationship between dolphin prey density and the number of conditioned dolphins, but that could be due to the small sample size (*n* = 10 years).

We found that conditioned dolphins had a higher probability of being injured compared with unconditioned animals. These injuries are likely to be the result of dolphins spending more time in close proximity to humans, boats and fishing gear, where they risk injury from collision with boats and entanglement in, hooking by and/or ingestion of fishing gear [27]. Wells *et al.* [31] showed that some of these injuries (e.g. fishing hooks embedded in the throat, goosebeak (modified larynx) or oesophagus, or fishing line wrapped around the goosebeak) often lead to death in dolphins. In Sarasota Bay, there are also reports of dolphins being killed by boat strikes [37], entanglement in fishing gear [38] and ingestion of fishing gear [30]. During 1993–2014, 83 dolphins (only 75 were included in this analysis) were observed with human-related injuries. Of these, 57 were attributed to entanglements, 17 to boat strikes and 16 to ingestion (seven individuals had multiple types of injuries either sequentially or at the same time; K.A.M. 2014, personal observation). In approximately 35% of cases, these injuries led to death or required rescue interventions (K.A.M. 2014, personal observation). Hence, conditioning of dolphins in Sarasota Bay may lead to a decrease in survival, which in turn could lead to population-level effects.

To prevent detrimental effects of food provisioning of bottlenose dolphins in Sarasota Bay, wildlife management needs to identify the factors contributing to animals becoming conditioned. In accordance with the findings of Donaldson *et al.* [16], we found that the probability of dolphins becoming conditioned was positively correlated with their association with already conditioned animals. This means that conditioning is, at least partly, a learned behaviour that is transmitted socially within the dolphin population. Horizontal learning of specialized foraging behaviours is well documented in dolphins, involving a variety of feeding patterns [49,50]. Further, it is possible that conditioning might also be spreading vertically through this population, similar to other foraging behaviours [51,52]. Social network analysis could be used to investigate this further and to quantify the relative importance of horizontal versus vertical transmission of conditioning in Sarasota Bay. While management can do little to prevent such socially learned behaviours from spreading, knowledge of how conditioning is transmitted through the population can help wildlife managers predict how quickly this behaviour will spread through the population, and help them to make a stronger case for stopping such behaviour at an early stage in a population.

Although we did not document a relationship between conditioning in dolphins relative to human exposure, it is logical that some level of exposure to human activities is necessary for conditioning to occur, because dolphins cannot successfully use unnatural foraging behaviours unless humans intentionally or unintentionally provision animals during such encounters. With human activities and dolphin home ranges varying in both space and time [45,53–55], it is expected that individual exposure of dolphins to human activities will also vary spatio-temporally. While we were able to investigate the effect of spatial and temporal variation in human exposure on dolphin conditioning separately, the data restricted us from combining these two effects. Alternatively, perhaps our index of human exposure (i.e. proportion of time spent in proximity to boats and fishing gear) is unrelated to the probability of dolphins engaging in human–dolphin interactions. Further studies are needed to find out how human exposure influences conditioning of dolphins in Sarasota Bay. This is important, because human activities are where management can act to reduce harmful human–dolphin interactions.

With the number of conditioned dolphins in Sarasota Bay increasing rapidly and with conditioned animals more likely to be injured and potentially killed by human activities, management actions and outreach are urgently needed. Although the US Federal Law has prohibited the feeding of free-ranging dolphins since 1991, illegal feeding interactions still occur in Sarasota Bay and elsewhere. A study, investigating the effect of education on the provisioning of dolphins in Sarasota Bay, found that a small number of people intentionally violate the Marine Mammal Protection Act by provisioning dolphins despite being aware that it is illegal [26]. Hence, following the recommendation of Cunningham-Smith *et al.* [26], we suggest that increased, well-publicized law enforcement efforts may be required to reduce the harmful food provisioning of dolphins in Sarasota Bay. A substantial amount of provisioning contributing to conditioning in this area comes from recreational fishing activities, where humans are often following regulations requiring them to release undersized or non-target catch. Focused outreach and cooperation with anglers will be an important component of preventing such unintentional provisioning. Ultimately, a combined approach to prevent direct and indirect provisioning is necessary, which includes education about the harmful consequences of these interactions, enforcement action against those observed violating applicable laws, cooperative monitoring with anglers and other user groups to better understand the spatio-temporal dynamics involved, and information about best practices to reduce adverse interactions.

Wildlife provisioning is increasing globally, both as an indirect consequence of human encroachment on natural habitats as well as intentional provisioning, the latter often associated with wildlife tourism [2,3]. This study shows that animals conditioned to food provisioning are at higher risk of injury and death from human interactions. Studies on other taxa, both marine and terrestrial, show similar results [10,19,22], highlighting that food provisioning has the potential to negatively impact wildlife populations. Conversely, other studies have shown that food provisioning can have positive effects on wildlife populations [6,8], by increasing individual survival and reproductive success [4,5,7]. The discrepancy between these studies highlights the complexity of this topic, and further suggests that the effects of wildlife provisioning are likely to be case specific. Therefore, rather than calling for an end to wildlife provisioning, we urge wildlife managers to apply a similar approach as the one used in this study, to investigate the potential fitness consequences of provisioning and, if management interventions are warranted (or regulations dictate), identify and reduce the driving factors leading to conditioning.

## Supplementary Material

The electronic supplementary material (ESM_F_Christiansen) contains the following:Description of data sets and variables used in analyses Figure S1. Dolphin sightings per month and year Figure S2. Dolphin conditioning as a function of COA estimated over different time periods Figure S3. Temporal trends in boat intensity, COA, dolphin prey CPUE and red tide concentration Table S1. Definitions of dolphin behaviours indicative of conditioning Table S2. Dolphin prey and red tide sampling effort Table S3. Description of variables in the “Data.COA.analyses.txt” data set Table S4. Description of variables in the “Data.injury.analyses.txt” data set Table S5. Description of variables in the “Data.year.analyses.txt” data set

## Supplementary Material

Data sets

## Data accessibility

The datasets supporting this article have been uploaded as part of the electronic supplementary material.
