# Supplementary material for: Food provisioning increases the risk of injury in a long-lived marine top predator
Source: R Soc Open Sci. 2016 Dec 21;3(12):160560. doi: 10.1098/rsos.160560 (PMC5210683; doi:10.1098/rsos.160560)
Supplement: The electronic supplementary material (ESM_F_Christiansen) contains the following:Description of data sets and variables used in analyses Figure S1. Dolphin sightings per month and year Figure S2. Dolphin conditioning as a function of COA estimated over different time periods Figure S3. Temporal tre [file rsos160560supp1.docx]

*Royal Society Open Science*

**Food provisioning increases the risk of injury in a long-lived marine top predator**

Fredrik Christiansen, Katherine A. McHugh, Lars Bejder, Eilidh M. Siegal, David Lusseau, Elizabeth Berens McCabe, Gretchen Lovewell & Randall S. Wells

Email: f.christiansen@murdoch.edu.au (F Christiansen)

# Electronic supplementary materials:

**-Description of data sets and variables used in analyses**

**-Figure S1.** Dolphin sightings per month and year

**-Figure S2.** Dolphin conditioning as a function of COA estimated over different time periods

**-Figure S3.** Temporal trends in boat intensity, COA, dolphin prey CPUE and red tide concentration

**-Table S1.** Definitions of dolphin behaviours indicative of conditioning

**-Table S2.** Dolphin prey and red tide sampling effort

**-Table S3.** Description of variables in the “Data.COA.analyses.txt” data set

**-Table S4.** Description of variables in the “Data.injury.analyses.txt” data set

**-Table S5.** Description of variables in the “Data.year.analyses.txt” data set

## Description of data sets and variables used in analyses

### Response variables

#### Conditioning

Year-round systematic photographic identification surveys of bottlenose dolphins (*Tursiops truncatus*) were carried out in Sarasota Bay and adjacent bays, sounds, and Gulf of Mexico waters within ~1 km of shore (figure 1) on a monthly basis (figure S1) between 1993 and 2014 [1]. Surveys were conducted during 10 days each month from small (6-7 m), outboard-powered vessels following standard routes through the study area, selected daily depending on conditions and areas previously covered, such that each part of the dolphin community’s range is surveyed at least three times each month [1–3]. When a dolphin group was sighted, the dorsal fins of the dolphins were photographed, from which individual dolphins could be identified [4]. These data were used to create a sighting history database of which individuals were observed interacting with each other (i.e. being sighted in the same group on the same day). For each sighting, the presence of human-dolphin interactions (patrolling, scavenging, depredation, begging, provisioning and fixed gear interactions, see Table S1 for definitions) were also recorded and associated to a specific dolphin ID [5,6]. Human-dolphin interactions were recorded opportunistically through 2006 (and coded retroactively from sighting notes) with codes for specific behaviours of interest added to the regular photographic identification survey field protocols in 2007.

#### Presence of injuries

The presence of human-related body injuries on dolphins, including fishing gear entanglement or ingestion and boat strike injuries, were recorded between 1984 and 2014 via direct observation during photographic identification surveys, as well as during examinations of animals during capture-release efforts for life-history, rescue operations, or health assessments, or upon necropsy evaluation. Injury types were categorized after Wells et al. [7]. For example, cases of fishing gear entanglement or ingestion were based upon observations of hooks, lures, monofilament or braided fishing line, or crab trap float lines present on or embedded in free-ranging animals or recovered carcasses, or from scarring patterns consistent with wounds from lines. Vessel strike cases were based on the presence of deep, parallel, evenly spaced cuts presumably caused by boat propellers or skegs and/or evidence of severe blunt force trauma.

### Variables affecting the probability of conditioning

#### Exposure to human activities (EXP)

The relative exposure to human activities (boats, crab pots and fishing lines) was estimated for individual dolphins in 2013 and 2014. First, the study area was divided into 1 km^2^ grid cells. The sighting data set was then used to link each sighting of an individual dolphin to a specific grid cell in the study area. Using this data, spatially explicit capture-recapture (SECR) models were then used to estimate the encounter probability of individual dolphins (the probability of sighting a specific individual per cell per minute of effort in each year), while accounting for spatiotemporal variations in sampling effort [8,9]. To quantify spatiotemporal variations in human intensity, the number of boats, crab pots and fishing lines were recorded within 50m of each dolphin sighting in 2013 and 2014. The total number of boats, fishing lines and crab pots was then summed in each cell per year. We then summed the number of dolphin sightings with potential human-dolphin interactions in each cell per year (i.e. the total number of dolphin sightings where a minimum of one boat, fishing line or crab pot was present). The total count of fishing gear (number of boats, crab pots and fishing lines) was then divided by the number of dolphin sightings with potential human-dolphin interaction. This produced a relative metric of human intensity (per sightings with human-interaction potential) for each cell per year. By multiplying the relative human intensity with the encounter probability of individual dolphins, and then summing this together across all cells, we obtained an estimate of the total exposure to human activities for each individual dolphin per year. Because boats, line and crab pots were only recorded throughout the year in 2013 and 2014, individual relative exposure was restricted to these two years.

#### Coefficient of Association with conditioned animals (COA)

To estimate the coefficient of association with conditioned animals (COA), we used sighting data of dolphin groups between 1993 and 2014. Each day was considered an independent sampling period. For each dolphin group sighting, the number of individuals in that group was identified using photo ID. The coefficient of association between all individuals within Sarasota Bay was then calculated between 1993 and 2014, using the half-weight association index (*AI*) [10]:

$$AI=\frac{x}{x+y_{AB}+\frac{1}{2}\left( y_{A}+y_{B} \right)}$$

where *x* is the number of sampling period (i.e. day) with individual *A* and *B* observed associated (i.e. in the same sighting), *y_A_* is the number of sampling periods with only individual *A* identified, *y_B_* is the number of sampling periods with only individual *B* identified and *y_AB_* is the number of sampling periods with individual *A* and *B* identified but not associated (both individuals observed in separate sightings within the same day).

The average coefficient of association of an individual to all conditioned dolphins in the population was then estimated [6]. To determine the COA for dolphins that became conditioned during the study, we used COA data for years before they became conditioned. Because we did not know the time period over which conditioning is transmitted between dolphins, we estimated the average COA over periods of one to five years prior to conditioning. For unconditioned animals, the average COA was estimated over one to five years before the end of the study (2014). Only individuals that had been sighted on at least 10 days during the study period were included in analysis. Unconditioned animals that died during the study period (n=44) were also removed from analysis.

#### Age and Sex

The age of 237 dolphins was either determined from longitudinal sighting histories for individuals known since birth (sighting histories for some individuals began as early as 1970) [2] or was estimated from examination of growth layer groups in teeth extracted during necropsy or under local anaesthesia during capture-release efforts [11]. The sex of 264 individual dolphins was determined either by direct observation or examination of the genital region, genetics, or, for some females, repeated observation with a dependent calf [2].

### Variables affecting the number of conditioned dolphins

#### Boat intensity

Temporal data on the annual number of registered boats (dealer, pleasure and commercial) in Manatee County and Sarasota County (figure 1) was obtained from the Florida Department of Highway Safety and Motor Vehicles between 2000 and 2014 (available at: http://www.flhsmv.gov/dmv/vslfacts.html). To investigate the effect of boat intensity on the number of conditioned dolphins, boat numbers were analysed separately (dealer, pleasure and commercial) as well as together, and also for the two counties separately and together.

#### Average coefficient of association with conditioned animals

To investigate yearly variation in number of conditioned dolphins, the average COA for all unconditioned animals for a given year was calculated.

#### Prey density

To assess the relative prey density in the study area, we conducted multispecies fish surveys using a 183 X 6.6m purse seine net with 2.54cm mesh from R/V Flip in shallow estuarine seagrass beds at water depths of 0.4–2.5m, following the methods of Gannon *et al.* [12]. Sampling occurred in the summer (June-September) months from 2004-2014. These fish surveys employed a random sampling design, with the overall the spatial extent of prey sampling designed to closely match the distributions of the resident dolphins sampled. All fishes captured in each seine set were identified to species and counted. Fish species considered to be dolphin prey included ladyfish (*Elops saurus*), pigfish (*Orthopristis chrysoptera*), pinfish (*Lagodon rhomboides*), spot (*Leiostomus xanthurus*), sheepshead (*Archosargus probatocephalus*), mullet (*Mugil* sp.), Gulf toadfish (*Opsanus beta*), spotted seatrout (*Cynoscion nebulosus*), scaled sardine (*Harengula jaguana*) and Atlantic thread herring (*Opisthonema oglinum*). These select species were considered to be commonly consumed dolphin prey based on previous research using stomach content analysis, molecular prey detection analysis, and prey selection [13–15]. For each year, the total catch of select dolphin prey species was divided by the fishing effort (total number of seine sets) to calculate the catch-per-unit-effort (CPUE) of select dolphin prey species, and used as a measure of relative dolphin prey density in the study area. A total of 223,980 dolphin prey fish were caught in 383 purse seine sets during the sampling period. Annual dolphin prey CPUE ranged from 245-998 individuals per set (table S2).

#### Red tide intensity

The relative severity of *Karenia brevis* red tide blooms, as measured by the number of weeks per year with *K. brevis* concentrations above 100,000 cells per litre, was included as a covariate to investigate the number of conditioned dolphins in the study area. Cell densities were sampled between 1987 and 2014 in Sarasota Bay by the Sarasota Dolphin Research Program (sampled twice at 10 fixed stations each month and once at each randomly sampled fish sampling site, 2005-2014), Mote Marine Lab’s Phytoplankton Ecology Program (sampled once daily at two fixed sampling stations and opportunistically, from 1987-2014), and the Florida Wildlife Research Institute’s (FWRI) red tide program (sampled opportunistically, from 1987-2014). Water sample collection and cell-counting procedures followed established protocols [16–18]. Surface water samples were collected in 20 ml glass scintillation vials, preserved with Utermöhl’s solution [19,20], and stored in darkness at room temperature until processing in the laboratory. The number of *K. brevis* cells in a 1 ml subsample was counted using an inverted Olympus CK40 microscope and then multiplied by 1,000 to obtain an estimate of the number of cells per litre [12]. For samples with high concentrations of *K. brevis,* 1:10 serial dilutions were performed using filtered water. A total of 11,591 *K. brevis* samples were analysed from 1987-2014. *K. brevis* cell densities ranged from 0-293,470,000 cells per litre during the study period (table S2). Data were then categorized by week following the FWRI’s red tide cell count categorizations (http://myfwc.com/research/redtide/statewide/). The total number of weeks with cell counts exceeding probable fish kill levels (i.e. >100,000 cells per litre) [12,21,22] within the resident Sarasota Bay dolphin community home ranges were calculated for each year. Red tide intensity has been found to correlate with high fish mortality [12,21,22], and may provide an indirect relative measure of prey availability in the area.

### References

1. Wells, R. S. 2014 Social structure and life history of common bottlenose dolphins near Sarasota Bay, Florida: Insights from four decades and five generations. In *Primates and cetaceans: Field research and conservation of complex mammalian societies* (eds J. Yamagiwa & L. Karczmarski), pp. 149–172. Tokyo, Japan: Springer.

2. Wells, R. S. 2009 Learning from nature: Bottlenose dolphin care and husbandry. *Zoo Biol.* **28**, 635–651.

3. Wells, R. S., Allen, J. B., Lovewell, G., Gorzelany, J., Delynn, R. E., Fauquier, D. A. & Barros, N. B. 2015 Carcass-recovery rates for resident bottlenose dolphins in Sarasota Bay, Florida. *Mar. Mammal Sci.* **31**, 355–368.

4. Hammond, P. S., Mizroch, S. A. & Donovan, G. P. 1990 Individual recognition of cetaceans: Use of photo-identification and other techniques to estimate population parameters. *Reports Int. Whal. Comm.* , 440.

5. Finn, H., Donaldson, R. & Calver, M. 2008 Feeding flipper: a case study of a human-dolphin interaction. *Pacific Conserv. Biol.* **14**, 215–225.

6. Donaldson, R., Finn, H., Bejder, L., Lusseau, D. & Calver, M. 2012 The social side of human-wildlife interaction: wildlife can learn harmful behaviours from each other. *Anim. Conserv.* **15**, 427–435.

7. Wells, R. S. et al. 2008 Consequences of injuries on survival and reproduction of common bottlenose dolphins (*Tursiops truncatus*) along the west coast of Florida. *Mar. Mammal Sci.* **24**, 774–794.

8. Pirotta, E., Thompson, P. M., Cheney, B., Donovan, C. R. & Lusseau, D. 2015 Estimating spatial, temporal and individual variability in dolphin cumulative exposure to boat traffic using spatially explicit capture-recapture methods. *Anim. Conserv.* **18**, 20–31.

9. Christiansen, F., Bertulli, C. G., Rasmussen, M. H. & Lusseau, D. 2015 Estimating cumulative exposure of wildlife to non-lethal disturbance using spatially explicit capture-recapture models. *J. Wildl. Manage.* **79**, 311–324.

10. Whitehead, H. 2008 *Analyzing animal societies: quantitative methods for vertebrate social analysis*. University of Chicago Press.

11. Hohn, A. A., Scott, M. D., Wells, R. S. & Sweeney, J. C. 1989 Growth layers in teeth from known-age, free-ranging bottlenose dolphins. *Mar. Mammal Sci.* **5**, 315–342.

12. Gannon, D. P., Berens McCabe, E. J., Camilleri, S. A., Gannon, J. G., Brueggen, M. K., Barleycorn, A. A., Palubok, V. I., Kirkpatrick, G. J. & Wells, R. S. 2009 Effects of *Karenia brevis* harmful algal blooms on nearshore fish communities in southwest Florida. *Mar. Ecol. Prog. Ser.* **378**, 171–186.

13. Barros, N. B. & Wells, R. S. 1998 Prey and feeding patterns of resident bottlenose dolphins (*Tursiops truncatus*) in Sarasota Bay, Florida. *J. Mammal.* **79**, 1045–1059.

14. Berens McCabe, E. J., Gannon, D. P., Barros, N. B. & Wells, R. S. 2010 Prey selection by resident common bottlenose dolphins (*Tursiops truncatus*) in Sarasota Bay, Florida. *Mar. Biol.* **157**, 931–942.

15. Dunshea, G., Barros, N. B., Berens McCabe, E. J., Gales, N. J., Hindell, M. A., Jarman, S. N. & Wells, R. S. 2013 Stranded dolphin stomach contents represent the free-ranging population’s diet. *Biol. Lett.* **9**, 20121036.

16. Lund, J. W. G., Kipling, C. & LeCren, E. D. 1958 The inverted microscope method of estimating algal numbers and the statistical basis of estimations of counting. *Hydrobiologia* **11**, 143–170.

17. Sournia, A. 1978 *Phytoplankton manual*. Paris: UNESCO.

18. Sellner, K. G., Doucette, G. J. & Kirkpatrick, G. J. 2003 Harmful algal blooms: causes, impacts, and detection. *J. Ind. Microbiol.* **30**, 383–406.

19. Utermöhl, H. 1958 Zur vervollkommnung der quantitativen phytoplankton-methodik. *Mitteilung Int. Vereinigung fuer Theor. unde Amgewandte Limnol.* **9**, 1–38.

20. Guillard, R. 1973 Division rates. In *Phycological methods* (ed J. Stein), pp. 289–311. Cambridge: Cambridge University Press.

21. Quick, J. A. & Henderson, G. E. 1975 Effects of *Gymnodinium breve* red tide on fishes and birds: a preliminary report on behavior, anatomy, hematology, and histopathology. In *Proceedings of the Gulf Coast regional symposium on diseases of aquatic animals*, pp. 85–115.

22. Landsberg, J. H. & Steidinger, K. A. 1998 A historical review of *Gymnodinium breve* red tides implicated in mass mortalities of the manatee (*Trichechus manatus latirostris*) in Florida, USA. In *Harmful algae* (eds B. Reguera J. Blanco M. L. Fernandez & T. Wyatt), pp. 97–100. Xunta de Galicia and Intergovernmental Oceanographic Commission, UNESCO.

## Figures

**Figure S1.** Number of dolphin sightings (indicated by colour scale) for each month and year during the study period. The number of dolphin sightings is also given by the numbers in the cells.


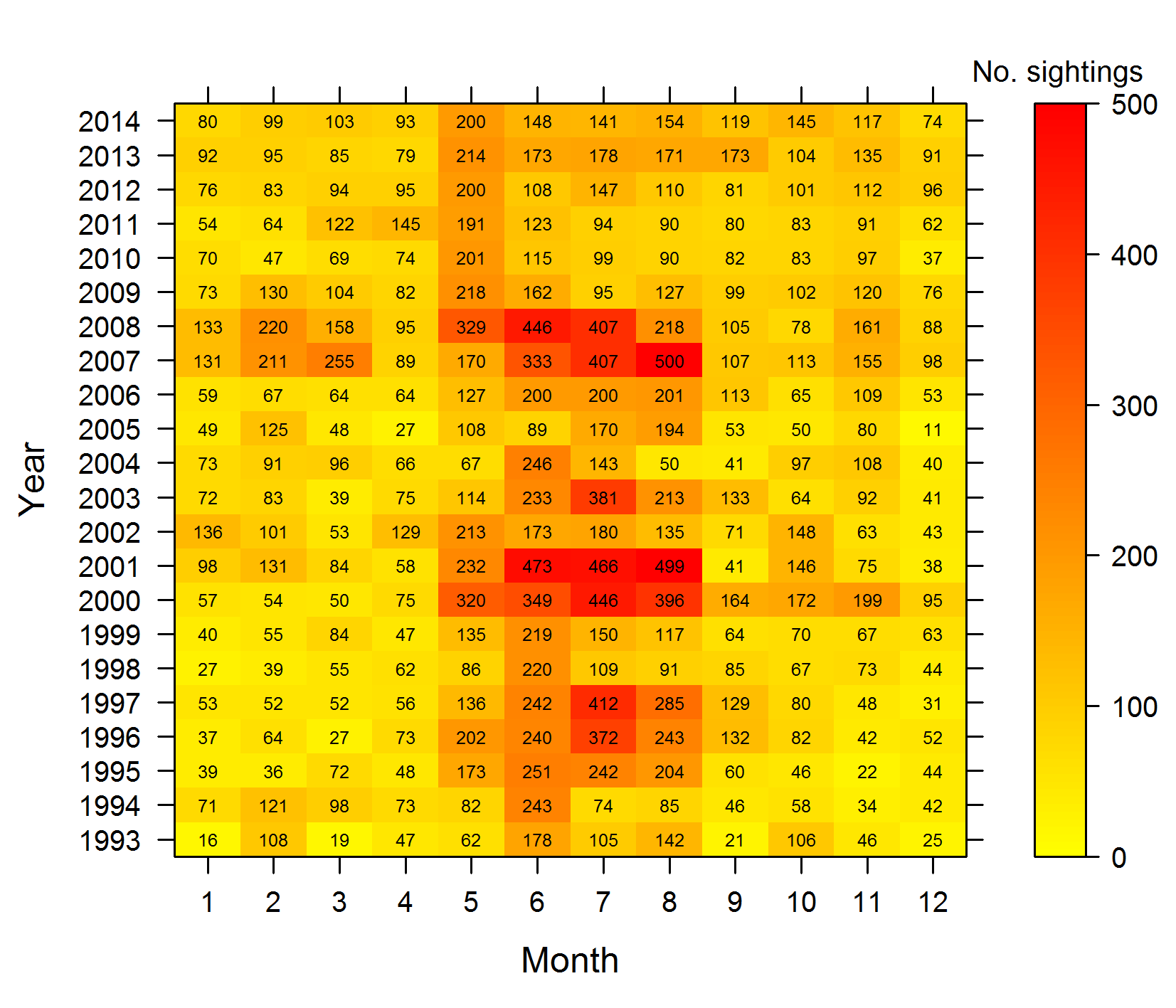


**Figure S2.** Probability of conditioning in bottlenose dolphins as a function of the coefficient of association with already conditioned animals (COA). The lines represent the fitted values of the generalized linear models, with the line types corresponding to the different time periods over which COA was estimated (between one to five years). The red line highlights the best fitting model (i.e. COA estimated over two years) based on model selection using Akaike’s Information Criterion (AIC_1 year_=413.0, AIC_2 years_=400.4, AIC_3 years_=424.1, AIC_4 years_=452.8, AIC_5 years_=469.5). n=524.**
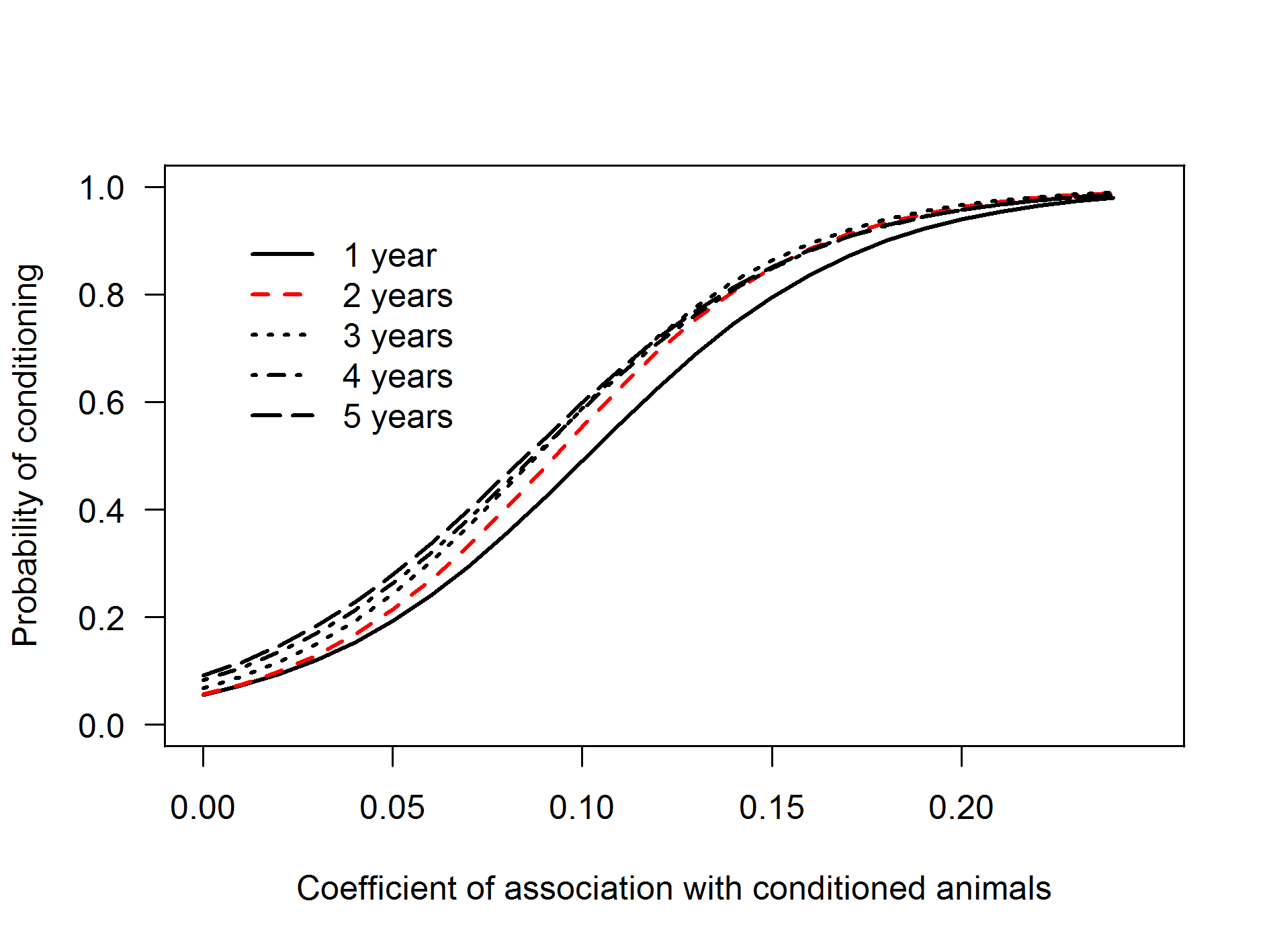
**

**Figure S3.** Temporal trends in (*a*) boat intensity, (*b*) coefficient of association with conditioned animals (COA), (*c*) catch-per-unit-effort (CPUE) of dolphin prey species and (*d*) number of weeks with high red tide concentrations (>100,000 *Karenia brevis* cells per litre). A cubic smoothing spline (black solid lines) was fitted to each variable, with the degrees of freedom set to five. n=10 years.

**
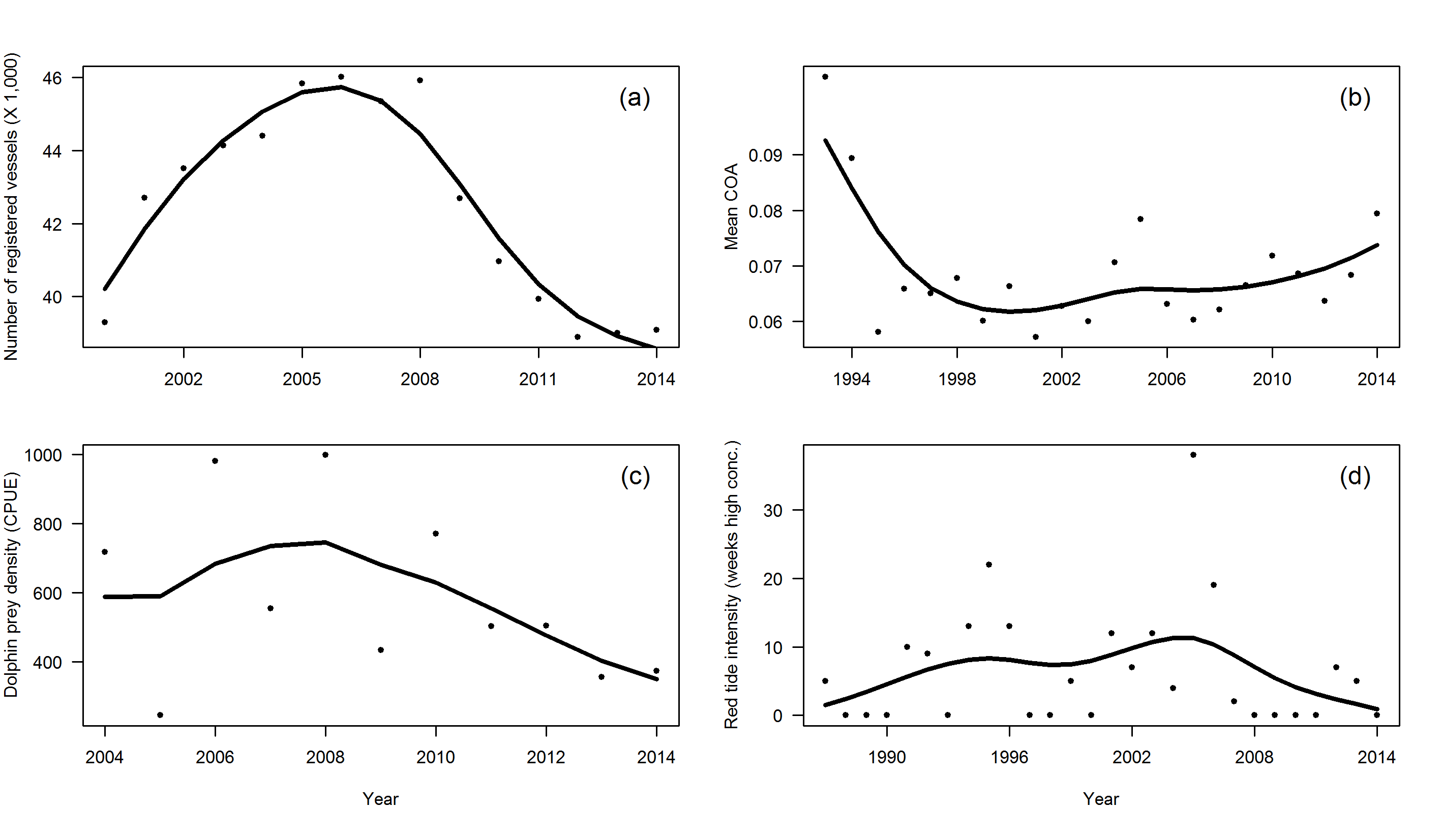
**

## Tables

**Table S1.** Definitions of dolphin behaviours indicative of conditioning

| Behaviour | Definition |
| --- | --- |
| Patrolling | Dolphin milling or travelling back and forth within 20m of boats, lines, or pier |
| Scavenging | Observed or probable feeding by dolphins on bait or catch throwback not on angler's line, when angler did not intend to feed dolphin |
| Depredation | Dolphin successfully taking and feeding on some or all of bait or catch from angler's line. Also includes unsuccessful or aborted depredation attempt or if it is not possible to determine the success |
| Begging | Dolphin behaviour(s) to elicit food from a person, such as bringing head out of the water and/or opening mouth at surface. Typically chin is out of the water |
| Provisioning | Dolphin intentionally being fed bait, catch or other item(s) by humans. May be directly dropped into mouth or thrown toward dolphin. Objects are released to dolphin. Includes humans trying to feed bait, catch or other item(s) to dolphins when it is unclear if the dolphins took the item |
| Fixed gear interactions | Repeated diving, milling, or probable feeding in close proximity to crab pots or other fixed fishing gear. May or may not include direct physical contact with a trap and/or its associated line and float |

**Table S2.** Total number of *Karenia brevis* samples, and sampling effort, total catch, and catch-per-unit-effort (CPUE) of selected dolphin prey species caught in seagrass habitat from 2004-2014 in Sarasota Bay, Florida. SD=standard deviation

| Year | No. *Karenia brevis* samples | No. seine sets | Total dolphin prey catch | CPUE | SD |
| --- | --- | --- | --- | --- | --- |
| 2004 | 297 | 16 | 11498 | 718.63 | 695.31 |
| 2005 | 743 | 24 | 5884 | 245.17 | 365.83 |
| 2006 | 1597 | 33 | 32403 | 981.91 | 1774.96 |
| 2007 | 1800 | 30 | 16624 | 554.13 | 599.61 |
| 2008 | 1500 | 40 | 39936 | 998.40 | 1109.62 |
| 2009 | 1313 | 40 | 17336 | 433.40 | 352.11 |
| 2010 | 994 | 40 | 30815 | 770.38 | 1147.79 |
| 2011 | 1007 | 40 | 20113 | 502.83 | 483.15 |
| 2012 | 909 | 40 | 20195 | 504.88 | 397.30 |
| 2013 | 1024 | 40 | 14228 | 355.70 | 353.37 |
| 2014 | 407 | 40 | 14948 | 373.70 | 311.76 |

**Table S3.** Description of variables in the “Data.COA.analyses.txt” data set

| Variable | Description |
| --- | --- |
| HI_Indiv | Classification of dolphins as either conditioned (1) or unconditioned (0) to food provisioning |
| COA.five.year | Average COA with conditioned animals over five years the last five years prior to conditioning (for conditioned dolphins) or before last estimate (for unconditioned dolphins) |
| COA.four.year | Same as above, but estimated over four years |
| COA.three.year | Same as above, but estimated over three years |
| COA.two.year | Same as above, but estimated over two years |
| COA.one.year | Same as above, but estimated over one year |

**Table S4.** Description of variables in the “Data.injury.analyses.txt” data set

| Variable | Description |
| --- | --- |
| HI_Injury | Classification of dolphins as either being injured (1) or not (0) as a result of human interactions |
| BirthYear | The year of birth for the dolphin |
| HI_Indiv | Classification of dolphins as either conditioned (1) or unconditioned (0) to food provisioning |
| Age | The age of the dolphin (in 2014) |

**Table S5.** Description of variables in the “Data.year.analyses.txt” data set

| Variable | Description |
| --- | --- |
| Year | The year |
| No.HI | The number of conditioned dolphins in a given year |
| Mean.COA | The mean COA with conditioned dolphins for all unconditioned dolphins in a given year |
| Median.COA | The median COA with conditioned dolphins for all unconditioned dolphins in a given year |
| CPUE.dolphin.prey | Catch per unit effort of dolphin prey in a given year |
| Red.tide.weeks | number of weeks per year with Karenia brevis concentrations above fish kill levels (>100,000 cells per litre) |
| Total.Total | The number of registered boats in Manatee County and Sarasota County in a given year |
